# Supplementary material for: Modeling glioblastoma heterogeneity as a dynamic network of cell states
Source: Mol Syst Biol. 2021 Sep 16;17(9):e10105. doi: 10.15252/msb.202010105 (PMC8444284; doi:10.15252/msb.202010105)
Supplement: Supplementary file 6 — Source Data for Figure 5 [file MSB-17-e10105-s004.zip › Figure5A_sourcedata/GSEA_3017/nosigdb_stateF.GseaPreranked.1622795015644/RICHARDS_NEFTEL_CELL_2019_AC.html]

Details for gene set RICHARDS\_NEFTEL\_CELL\_2019\_AC[GSEA]

|  || Dataset | state33017 |
| Phenotype | NoPhenotypeAvailable |
| Upregulated in class | na\_pos |
| GeneSet | RICHARDS\_NEFTEL\_CELL\_2019\_AC |
| Enrichment Score (ES) | 0.7673228 |
| Normalized Enrichment Score (NES) | 3.551935 |
| Nominal p-value | 0.0 |
| FDR q-value | 0.0 |
| FWER p-Value | 0.0 |
Table: GSEA Results Summary

  

Fig 1: Enrichment plot: RICHARDS\_NEFTEL\_CELL\_2019\_AC      
 Profile of the Running ES Score & Positions of GeneSet Members on the Rank Ordered List

  

| PROBE | GENE SYMBOL | GENE\_TITLE | RANK IN GENE LIST | RANK METRIC SCORE | RUNNING ES | CORE ENRICHMENT || 1 | MT3 |  |  | 1 | 2.229 | 0.0765 | Yes |
| 2 | CLU |  |  | 5 | 1.852 | 0.1390 | Yes |
| 3 | SPARCL1 |  |  | 6 | 1.804 | 0.2013 | Yes |
| 4 | ATP1B2 |  |  | 9 | 1.759 | 0.2611 | Yes |
| 5 | HEPN1 |  |  | 11 | 1.554 | 0.3142 | Yes |
| 6 | HOPX |  |  | 14 | 1.345 | 0.3597 | Yes |
| 7 | CST3 |  |  | 15 | 1.273 | 0.4037 | Yes |
| 8 | S100B |  |  | 20 | 1.167 | 0.4421 | Yes |
| 9 | RAMP1 |  |  | 24 | 1.069 | 0.4777 | Yes |
| 10 | SLC1A3 |  |  | 26 | 1.058 | 0.5137 | Yes |
| 11 | PLTP |  |  | 35 | 1.020 | 0.5453 | Yes |
| 12 | F3 |  |  | 41 | 0.978 | 0.5768 | Yes |
| 13 | EDNRB |  |  | 44 | 0.957 | 0.6090 | Yes |
| 14 | GFAP |  |  | 47 | 0.953 | 0.6409 | Yes |
| 15 | SPARC |  |  | 52 | 0.929 | 0.6712 | Yes |
| 16 | MLC1 |  |  | 58 | 0.896 | 0.6999 | Yes |
| 17 | AGT |  |  | 72 | 0.820 | 0.7223 | Yes |
| 18 | AQP4 |  |  | 84 | 0.789 | 0.7446 | Yes |
| 19 | PON2 |  |  | 93 | 0.764 | 0.7673 | Yes |
| 20 | TSPAN7 |  |  | 148 | 0.671 | 0.7662 | No |
| 21 | BCAN |  |  | 302 | 0.510 | 0.7149 | No |
| 22 | PTPRZ1 |  |  | 310 | 0.505 | 0.7292 | No |
| 23 | GATM |  |  | 375 | 0.480 | 0.7169 | No |
| 24 | PRCP |  |  | 426 | 0.460 | 0.7103 | No |
| 25 | TTYH1 |  |  | 457 | 0.448 | 0.7122 | No |
| 26 | HEY1 |  |  | 496 | 0.436 | 0.7102 | No |
| 27 | GPM6B |  |  | 553 | 0.413 | 0.6992 | No |
| 28 | PMP2 |  |  | 562 | 0.411 | 0.7098 | No |
| 29 | RAB31 |  |  | 580 | 0.404 | 0.7161 | No |
| 30 | FABP7 |  |  | 701 | 0.371 | 0.6749 | No |
| 31 | PCDHGC3 |  |  | 845 | 0.337 | 0.6222 | No |
| 32 | METTL7B |  |  | 1704 | -0.320 | 0.2471 | No |
Table: GSEA details [plain text format]

  

Fig 2: RICHARDS\_NEFTEL\_CELL\_2019\_AC: Random ES distribution      
 Gene set null distribution of ES for **RICHARDS\_NEFTEL\_CELL\_2019\_AC**
